# Advances and challenges in multiscale biomolecular simulations: artificial intelligence‐driven paradigm shift

**DOI:** 10.1002/qub2.70024

**Published:** 2025-12-03

**Authors:** Wenfei Li, Wei Wang

**Affiliations:** ^1^ Department of Physics National Laboratory of Solid State Microstructure Nanjing University Nanjing China

**Keywords:** artificial intelligence, biomolecules, molecular dynamics, protein dynamics

## Abstract

Molecular simulation techniques have become an invaluable tool for elucidating the fundamental principles of life at the molecular level. After nearly five decades of development, biomolecular simulations have evolved to enable the quantitative characterization of complex biomolecular events, such as protein folding, conformational dynamics, and protein–protein interactions. These advancements have significantly influenced both fundamental and applied research. In recent years, the integration of machine learning, particularly deep learning algorithms, has further driven innovation in this field. This perspective aims to discuss the latest advancements in biomolecular simulation techniques and to explore emerging applications, development trends, and major challenges in biomolecular dynamics simulations.

## 1 FROM STATIC STRUCTURES TO FUNCTIONAL DYNAMICS

Life processes at the molecular level primarily manifest as the assembly, motion, and interactions of biomolecules. Consequently, deciphering the principles that underlie biological function demands not only high‐resolution three‐dimensional structures but also a mechanistic understanding of the dynamic processes that drive activity. Recent advances powered by an ever‐growing repository of protein structures and breakthroughs in artificial intelligence (AI) have enabled platforms such as AlphaFold2 to achieve transformative progress in static protein structure prediction, effectively resolving the long‐standing structure prediction challenge [1, 2, 3, 4, 5, 6]. However, experimental characterization of biomolecular dynamics remains significantly underdeveloped compared to static structure analysis. This deficiency has created a critical shortage of dynamics data, severely limiting AI applications in biomolecular dynamics prediction. Molecular simulation techniques, particularly molecular dynamics (MD), are garnering increased attention as powerful tools for characterizing biomolecular dynamics [7, 8, 9, 10, 11].

## 2 ACCURACY‐EFFICIENCY BOTTLENECK IN BIOMOLECULAR SIMULATIONS

**FIGURE 1:**
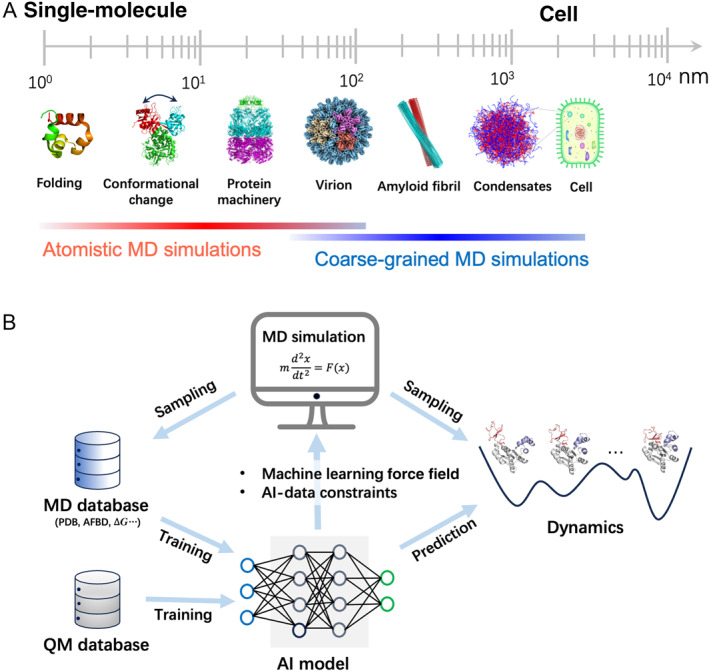
Schematic for the multiscale challenge of biomolecular simulations and its solution by AI‐based methods. (A) Multiscale challenge of biomolecular simulations. (B) Modeling biomolecular dynamics by integrating molecular simulations and AI. Three strategies are illustrated: MLFF construction for high‐accuracy MD simulations; AI‐derived constraints guiding MD simulations; and AI models trained on MD data to predict protein dynamics. AI, artificial intelligence; MD, molecular dynamics; MLFF, machine learning force field.

Classical MD simulations generate atomistic trajectories of biomolecules, and thus the associated kinetic and thermodynamic properties, by integrating Newton’s equations with empirical force fields [7, 12, 13]. However, the vast number of degrees of freedom and complex high‐dimensional energy landscapes inherent to biomolecules render exhaustive sampling exceptionally challenging [14, 15, 16]. Functionally critical processes in proteins and other biomolecules span multiple spatiotemporal scales (Figure 1A) [17, 18]. Current atomistic MD simulations with enhanced sampling algorithms can access the timescales involved in the folding and conformational changes of proteins with moderate sizes. However, for protein dynamics involving collective behaviors of multiple biomolecules, such as the assembly and functional motions of large protein machineries, amyloid fibril aggregation, and biomolecular liquid–liquid phase separation (LLPS), which operate across much larger spatiotemporal scales, conventional atomistic simulations become computationally prohibitive. To overcome this computational barrier, coarse‐grained (CG) models [15, 19, 20, 21, 22, 23], in which a group of atoms are simplified into single virtual particles, are introduced. Although the CG approximation provides a solution to overcome the efficiency bottleneck of MD simulations, developing CG force fields that faithfully capture essential features of biomolecular interactions and dynamics remains profoundly difficult. Thus, enhancing the accuracy and efficiency constitutes a core mission and longstanding challenge in biomolecular simulations. Recently, AI‐powered algorithms have emerged as novel pathways to address the accuracy–efficiency bottleneck (Figure 1B) [24, 25, 26, 27].

## 3 AI‐DRIVEN MOLECULAR SIMULATIONS WITH QUANTUM MECHANICAL ACCURACY

The functional motions and interactions of biomolecules depend on their microscopic electronic structures, which in principle demand a quantum mechanical (QM) treatment. However, conventional QM methods remain computationally prohibitive, limiting their application to small systems and short timescales. All‐atom molecular force field model enhances computational efficiency by applying Born–Oppenheimer approximation, frozen‐electron ground‐state assumption, and empirical parametrization, but at the cost of reduced accuracy relative to ab initio approaches. Bridging this gap has long been a central goal in computational biophysics.

Deep neural networks provide powerful parameterization schemes without assuming predefined functional forms. By training machine learning force fields (MLFFs) on QM reference datasets, these models directly predict energies and forces from atomic coordinates without explicit electronic‐structure calculations, achieving high‐efficiency biomolecular computations at QM accuracy. In recent years, AI‐driven quantum‐accuracy simulation methods have made rapid progress [26, 28, 29, 30, 31], enabling efficient and high‐precision single‐point energy evaluations, nuclear magnetic resonance (NMR) chemical shift predictions, protein folding free‐energy estimation, and pKa calculations. Remarkably, the AI^2^BMD framework developed recently by Wang and colleagues has achieved QM‐level simulations of small‐protein folding and improved estimation of thermodynamic properties [26].

A key requirement for MLFFs is extensive QM training data, but high‐throughput ab initio calculations on entire proteins remain intractable. This issue can be resolved by employing a divide‐and‐conquer strategy, that is, decomposing the large protein system into QM‐treatable and overlapping fragments. These well‐established fragmentation methodologies can be directly utilized to construct QM databases in training MLFFs [32, 33, 34]. It is worth noting that MLFFs leverage neural networks’ exceptional fitting capacity but lack physically explicit energy functions, inherently limiting transferability. Expanding training datasets and establishing rigorous validation metrics are therefore essential tasks in future development. Additionally, reasonably modeling the key physical processes involving quantum effects, for example, catalytic reactions, charge transfer, and protonation dynamics of biomolecules, within the MLFF framework, are still challenging tasks.

## 4 AI‐DRIVEN MULTISCALE BIOMOLECULAR SIMULATIONS

Due to the inherent multiscale nature of proteins and other biomolecules, even the all‐atom MD simulations using empirical force fields are limited to small spatiotemporal scales (Figure 1A). For large‐scale biomolecular assemblies and functional motions, CG approximations become essential to overcome computational complexity. In CG models, a group of atoms is mapped onto single virtual particles, drastically reducing system degrees of freedom and accelerating simulations. Unlike all‐atom force fields parameterized against QM data, CG force fields need to reproduce conformational ensemble properties of higher‐dimensional molecular systems within reduced reaction coordinates. Consequently, CG force fields represent the potential of mean force projected onto CG reaction coordinates, including not only energetic terms but also contributions from conformational entropy of integrated fine‐grained degrees of freedom. This makes the development of CG force fields extremely challenging and requires much more complex functional forms.

The success of MLFFs has provided novel solutions for constructing CG force fields attributing to the powerful function representation capability of deep neural networks [29, 35]. Currently, two primary strategies are employed to develop CG force fields using MLFFs, including the bottom–up and top–down strategies [35, 36, 37]. The bottom–up strategy directly trains the CG MLFFs using energy or force data from QM calculations or all‐atom simulations. For example, CGNet and its more recent version CGSchNet utilize the force‐matching technique to extract force field parameters, that is, by optimizing CG MLFFs by matching forces on CG particles computed from all‐atom models [12, 35]. CG MLFFs derived through this bottom–up method have been successfully applied to the simulations of protein folding, which can reasonably reproduce the free energy surfaces and other statistical properties from all‐atom simulations. In contrast, the top–down strategy optimizes deep neural network parameters to fit the global observables of biomolecules, for example, root mean square deviation from reference protein structures [36]. In addition to learning and representing the CG force field, generative AI models have also been used to reconstruct the all‐atom structures from the CG structures [29] and to generate conformational ensembles for disordered proteins [38].

Because of the complex functional forms and limited transferability inherent to CG force‐field models, the development of CG MLFFs requires more comprehensive conformational space coverage in training datasets. However, databases derived from atomistic MD simulations in the training of CG MLFFs inevitably encounter inadequate sampling. This challenge cannot be resolved simply by scaling up conventional atomistic MD simulations. A promising solution involves implementing adaptive iterative multiscale simulations [39, 40], in which the CG model and the atomistic model are utilized concurrently, and the CG MLFFs are improved with the iteration. The adaptive iteration is designed so that the conformational sampling is always performed by CG simulations, whereas energy evaluations are based on all‐atom force fields. Early multiscale simulations have demonstrated the potential of this adaptive optimization methodology in solving the accuracy–efficiency bottleneck in biomolecular simulations [16, 18, 39].

## 5 INTEGRATIVE MOLECULAR DYNAMICS SIMULATIONS WITH AI‐DERIVED DATA

The transferability and accuracy of biomolecular force fields are not always consistent. To meet the transferability requirement, the results based on conventional biomolecular force fields often exhibit deviation from experimental data when applied to specific protein systems. On the other hand, substantial low‐resolution experimental data have been accumulated, including single‐molecule FRET, small‐angle X‐ray scattering, hydrogen‐deuterium exchange, infrared spectroscopy, and NMR [41, 42, 43, 44]. Integrating such multimodal experimental constraints into MD simulations through machine learning and other data‐driven algorithms significantly enhances simulation accuracy for target proteins [45, 46, 47, 48, 49]. This kind of integrative MD simulations demonstrates particular strengths in characterizing dynamic structural information in biomolecular events and has gained widespread applications. Recent advances in AI have substantially expanded biomolecular data dimensions. AlphaFold2 exemplifies this progress by delivering not only static structure predictions but also auxiliary metrics including pLDDT, predicted aligned error (PAE), and residue contact maps. These AI‐derived outputs reveal intrinsic structural and dynamic properties, such as structural order, local flexibility, and long‐range interaction signatures. These high‐quality AI‐derived data possess comparable values to experimental data and can be used to effectively constrain molecular simulations. In recent studies, the PAE data have already been used to dictate the molecular simulations with the elastic network model [50], and the simulation results can provide improved modeling of the protein flexibility and low‐frequency mode of functionally relevant collective motions. By integrating AlphaFold‐predicted residue contact probability matrix into CG simulations, researchers from Cambridge university substantially improved the conformational ensemble modeling of intrinsically disordered proteins (IDPs) [51]. Such methodologies applying system‐specific constraints to transferable force field models represent a balanced solutions that simultaneously address generality requirements and computational accuracy demands.

## 6 MOLECULAR DYNAMICS POWERED AI‐GENERATION OF PROTEIN CONFORMATIONAL ENSEMBLES

The above discussions have highlighted the critical role of AI in advancing molecular simulation techniques. It is noteworthy that conformational ensembles and other dynamic data generated through molecular simulations can conversely serve as valuable training resources for AI models, particularly in protein dynamics predictions. With the success of static protein structure prediction, protein dynamics prediction becomes the new focus of research efforts [52, 53, 54, 55, 56, 57, 58, 59, 60, 61, 62]. However, training AI model for dynamics prediction is much more challenging due to the lack of experimental data. The prevailing strategy to address this gap involves leveraging MD simulation data as training sets. Recently, such simulation‐derived data are increasingly becoming primary reference sources for training models that predict multi‐conformational states and generate conformational ensembles, including diffusion‐based and flow‐based generative models [38, 63, 64, 65, 66]. These models often need to be pretrained first on AlphaFold database and PDB structures, then fine‐tuned based on MD simulations and even protein stability measurements. Incorporating energy information and other physical constraints in training the model can also improve the prediction [63, 65, 66, 67].

In addition to MD‐powered AI models, other strategies have also been developed to predict protein dynamics based on AI framework. For instance, by integrating the biophysical principle of protein dynamics into the AlphaFold2 structure prediction pipeline [68, 69, 70, 71], it is possible to generate not only multiple conformations of allosteric proteins but also the pathways of their conformational motions [72]. By training a deep‐learning model with the transition state structures extracted from large scale CG simulations, one can also predict the pathways of protein conformational changes [73].

## 7 EXPANDING THE APPLICATION CUTTING EDGE OF BIOMOLECULAR SIMULATIONS

The development in the field of biomolecular simulations is not only reflected in the technical aspect but also in the application aspect. In fact, the advancement of biomolecular simulation technology is constantly expanding its application boundaries. Previous biomolecular simulations mainly focused on proteins, nucleic acids, and phospholipid molecules. Recently, the key role of post‐translational modifications of biological macromolecules, particularly glycosylation, in structural and functional dynamics has attracted attention. For instance, it has been discovered that the sugar chain of the spike protein of SARS‐CoV‐2 can assist in its immune escape [74]. In addition, glycosylation modification can regulate the LLPS and amyloid aggregation of proteins related to neurodegenerative diseases [75]. The dynamic nature of the sugar chain makes glycosylation difficult to characterize with traditional structural biology techniques, and it is thus called the “dark matter” in structural biology. Naturally, MD simulations are used to assist in the structural biology characterization of glycosylation. For instance, based on the sugar chain conformation database constructed by massive MD simulation, significant progress has been made in the predictions of glycosylation sites, sugar abundance, and glycoprotein conformations. Accordingly, tools such as GlycoSHIELD and GlycoSHAPE have been developed [75, 76]. With the development of glycosylation force fields and molecular simulation techniques, molecular dynamics will play an increasingly important role in explaining the molecular mechanism related to the structure and function of glycosylated proteins, providing key tools for the study of biological dark matter.

In addition to the research objects, the spatial scale of the simulation system is also expanding. For instance, the recent development of CG biomolecular force fields has made it possible to conduct high‐throughput simulations of IDPs. Consequently, the application of biomolecular simulations has been extended to the proteomic scales. Using the CALVADOS2 force field, researchers from the University of Copenhagen simulated tens of thousands of AlphaFold2‐predicted IDP regions across the human genome [20], revealing location‐ and function‐dependent patterns in IDP conformational compactness and linking cell‐level functions to the conformational properties of protein molecules. In addition, the accessible sizes of biomolecular simulation systems are also constantly expanding. For instance, by using the CG model, molecular simulations of LLPS systems containing thousands of protein chains can be achieved. These large scale simulations enable quantitative characterization of two‐phase coexistence curves, droplet fusion kinetics, and microphase separation [10, 21, 77, 78, 79], which is otherwise difficult to characterize in experiments. All‐atom MD simulations have also been applied to study the detailed dynamics and interaction characteristics of biological condensates at short time scales [10, 78]. In addition, molecular simulation works at the cytoplasmic and full‐virus scales are also constantly emerging [9, 80, 81].

## 8 DATA SHARING AND SOFTWARE DEVELOPMENT IN BIOMOLECULAR SIMULATIONS

In the era of big data, the value of biomolecular simulation data is becoming increasingly prominent, and data sharing and open access have received significant attention. Recently, over 100 biomolecular simulation experts co‐authored a paper in *Nature Methods* advocating FAIR principles (findable, accessible, interoperable, reusable) for biomolecular data sharing [82]. In line with the FAIR principle, depositing MD trajectory data alongside publications has become standard practice in recent years [83]. Platforms such as CERN’s Zenodo now host a vast amount of MD trajectory files. The e‐Science Center at Nanjing University also offers data‐sharing services for biomolecular simulations, along with other scientific computing supports. These biomolecular simulation data not only provide valuable insights into complex biomolecular dynamics but also become an important data source for various applications based on data‐driven AI technology. How to share MD data more efficiently and smoothly is worth the efforts in future studies.

In tandem with methodological innovations, MD simulation software has also rapidly evolved [84]. In addition to those established packages (e.g., GROMACS, AMBER, CHARMM, NAMD, and GENESIS) [85, 86, 87, 88, 89], open‐source frameworks, such as CafeMol, OpenMM, and its derivatives designed to facilitate GPU acceleration, CG simulations, and MLFFs are becoming popular [90, 91, 92, 93, 94, 95]. Future progress requires deeper integration of simulation and structural prediction tools within unified open‐source ecosystems, exemplified by platforms such as MindSpore SPONGE [96]. Meanwhile, advances in quantum computing and specialized hardware are expected to bring transformative breakthroughs for the biomolecular modeling field.

## AUTHOR CONTRIBUTIONS

**Wenfei Li**: Writing—original draft; writing—review and editing; visualization; funding acquisition. **Wei Wang**: Project administration; writing—review and editing; funding acquisition.

## CONFLICT OF INTEREST STATEMENT

The authors declare no conflicts of interest.

## ETHICS STATEMENT

This perspective article does not contain the studies related to human or animal subjects.

## Data Availability

This work does not produce any new data.
